# Effects of Medicinal Plants and Flavonoids on Parkinson’s Disease: A Review on Basic and Clinical Evidences

**DOI:** 10.34172/apb.2021.026

**Published:** 2020-07-01

**Authors:** Mohammad Reza Khazdair, Majid Kianmehr, Akbar Anaeigoudari

**Affiliations:** ^1^Cardiovascular Diseases Research Center, Birjand University of Medical Sciences, Birjand, Iran.; ^2^Esfarayen Faculty of Medical Sciences, Esfarayen, Iran.; ^3^Department of Physiology, School of Medicine, Jiroft University of Medical Sciences, Jiroft, Iran.

**Keywords:** Parkinson's disease, Neurotoxicity, Medicinal plants, Flavonoids

## Abstract

Parkinson’s disease (PD) is a neurodegenerative disorder which is characterized by typical symptoms including gradual progressive muscle rigidity, tremor and loss of motor skills. Although there is no definitive cure for PD, the extract of some medicinal plants and their ingredients have been suggested to relieve its symptoms and to prevent disability in patients. This review is focused on therapeutic effects of some medicinal plants and their ingredients on PD. The findings presented in this review were collected from experimental and clinical studies in databases including PubMed, Web of Science and Google Scholar until the end of May 2019. The keywords "neurotoxicity " or "Parkinson’s disease" or "neuroprotective" and "Medicinal plants" and "Flavonoids" were searched. Based on the results of animal and clinical studies, the extract of medicinal plants and their components which are discussed in this review have neuro-protective effects against PD. These protective properties mainly are mediated through inhibition of dopamine metabolizing enzymes, reduction oxidant markers, increase of antioxidant agents and suppression of neuro-inflammation.

## Introduction

Parkinson’s disease (PD) is considered as the most common neuronal destructive disease after Alzheimer’s disease (AD).^1^ This neurodegenerative disorder results from progressive damage in dopamine secreting cells in substantia nigra.^2^ Oxidative stress and neuro-inflammation have been recognized as key causes in dopaminergic neurons death in varous forms of PD.^3^ Researchers have been suggested that overload of reactive oxygen species (ROS) followed by brain ischemia can cause neurotoxicity resulting in PD.^4^ In addition, the contribution of nuclear factor κB (NF-κB), an effective key factor in expression of pro-inflammatory cytokines, to neuronal death in PD has been understood.^5,6^ The harmful impact of inflammatory mediators including tumor necrosis factors-α, interlukin (IL)-1β and IL-6, oxygen free radicals and inducible nitric oxide synthase on dopaminergic cells in substantia nigra pars compacta has been also ducumented.^7,8^ The drugs used for the cure of PD such as levodopa (L-dopa) and monoamine oxidase B (MAO_B_) inhibitors and dopamine agonists mdulate the brain dopamine content or trigger intracellular signalings through activating the dopamine receptors.^9^ Anticholinergic drugs have been also suggested to have anti-parkisonian effects.^10^ These medications have benefucial effects on rgidity and tremor in PD patients.^11^ In addition, antioxidant and anti-inflammatory agents have been shown to play a vital role in survival of neurons and alleviation of PD syptomes.^12,13^ Recently, the strong neuro-protective effect of medicinal plants extracts and phytochemicals in reduction of PD signs due to anti-oxidant and anti-inflammatory properties has been heighlited in various studies.^14-16^ Phytochemicals such as thymoquinone (TQ), crocin, curcumin and polyphenols have been shown to have cosidarable protective effects on nervous system via moulation oxidative stress and inflammatory responses.^17-20^ Therefore, the present review was aimed to investigate the therapeutic effects of medicinal plants and ingredients on PD.

## Methods

The data narrated in our review were assembled from databases PubMed, Web of Science and Google Scholar until the end of May 2019. Data consist of animal and clinical researches. Letter to the editors and non-English language articles were not considered.

### Mucuna pruriens

**Figure 1:**
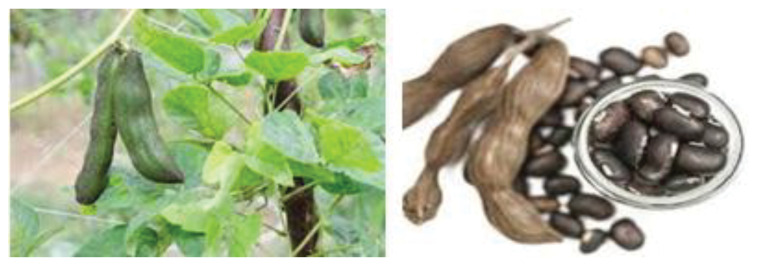
* Mucuna pruriens.*

*Mucuna pruriens* from the Fabaceae family has been used in Indian traditional medicine for curing diseases such as PD (see Figure 1).^21^ One of the principal constitutes of this plant is L-dopa.^22^ The administration of food endocarp of *M. pruriens* seeds (5 g/kg) combined with carbidopa (50 mg/kg) had better effect than L-dopa in the test of free contralateral rotation induced by 6-hydroxydopamine (6-OHDA) in mice.^23^ *M. pruriens* seeds extract (400 mg/kg) also applied a significant anti-Parkinson effect in rats.^24^ Treatment with* M. pruriens* powder (2.5 or 5 g/kg/d) remarkably elevated the endogenous level of L-dopa, dopamine, norepinephrine and serotonin in the substantia nigra in 6-OHDA-induced PD rat model.^25^

The HP-200 is a commercial preparation derived from *M. pruriens* in Ayurveda which has been proponed to have anti-Parkinson effects. The impact of *M. pruriens* endocarp in form of HP-200 (2.5, 5 or 10 g/kg/d) on the content of monoaminergic neurotransmitters in the different areas of the rats’ brain such as substantia nigra, striatum, cortex and hippocampus was pursued. Based on the results, this form of *M. pruriens* noticeably elevated the dopamine concentration in brain cortex of rats. Data also showed that this drug did not affect the concentration of L-dopa, dopamine, serotonin and norepinephrine in the nigrostriatal pathway of rats. These findings emphasizes that the anti-Parkinson properties of *M. pruriens* endocarp may be mediated via phytochemicals other than L-dopa or it likely can amplify the L-dopa effects.^26^

The clinical and pharmacokinetics effect of L-dopa followed by two doses of *M. pruriens* seeds powder** (**15 and 30 g**)** was assessed and compared with single doses of standard L-dopa/carbidopa (LD/CD) (200/50 mg) in PD patients. The results revealed that 30 g of *M. pruriens* seeds powder formulation possesses a marked quantity of L-dopa. This property was accompanied with the decreased duration without change of dyskinesia intensity in L-dopa response with respect to standard dose of this drug. These results suggest that this important and natural resource of L-dopa can possess helpful effects on L-dopa preparation in long lasting management of PD patients.^27^ Oral administration of *M. pruriens* powdered seeds (15 to 40 g) showed symptomatic control with the 4.5 to 5.5% of L-dopa in 33 patients with PD in a clinical trial.^28^ In the case report, carbidopa significantly led to the betterment of motor activities in a 48-year-old Parkinson woman when it was added to *M. pruriens*. This finding confirms that usage of a dopa-decarboxylase inhibitor to *M. pruriens* can be helpful for management of PD patients who are not interested to begin L-dopa.^29^ The protective effects of *M. pruriens* on PD have been presented in Table 1.

**Table 1:** The protective effect of *Mucuna pruriens* on PD

**Doses used**	**Model of study**	**Effects**	**Ref.**
5 g/kg + carbidopa	Mice	Improvement in the test of free contralateral rotation induced by 6‐OHDA compared with L-dopa	23
400 mg/kg,	Rat	Significant anti-Parkinson effect	24
2.5 or 5 g/kg/d	Rat	Restoration of endogenous neurotransmitters such as, L-dopa, dopamine, norepinephrine and serotonin content in the substantia nigra	25
2.5, 5 or 10 g/kg/d of Endocarp form of HP-200	Rat	Elevation of the dopamine concentration in brain cortex.	26
30 g	Human	Induction of marked quantity of L-dopa accompanied with the decreased duration without change of dyskinesia intensity in L-dopa response with respect to standard dose of this drug	27
15 to 40 g	Human	Symptomatic control with the 4.5% to 5.5% of L-dopa	28

### Vicia faba L.

**Figure 2:**
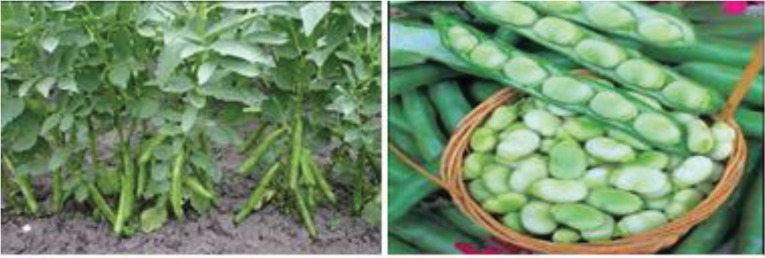
* Vicia faba* L.

*Vicia faba* which is known as broad beans, horse beans, or field beans is used as food for many years in the Mediterranean area, India, Pakistan, and China (Figure 2). The seeds of this rich natural source of L-dopa are full of proteins, carbohydrates, fiber, and vitamins.^30^ In a report, the usage of *V. faba* in remedy of PD patients resulted in ameliorating motor activity. This effect was comparable with treatment by L-dopa (125 mg) plus carbidopa (12 5 mg). It was indicated that the severity of dyskinesia in *V. faba*-treated three patients was similar to those of treated by L-dopa. In addition, plasma content of L-dopa significantly was high after *V. faba* ingestion in PD patients.^31^ In another study, administration of *V. faba* (250 g cooked) after 12 hours off medication in healthy volunteers (n=5) and PD patients (n=6) improved the clinical signs and enhanced the plasma content of L-dopa.^32^ Single dose of *V. faba* mixture (200 g) plus carbidopa in six PD patients showed the increased duration of motor response to *V. faba* compared with L-dopa medication. The prolonged motor response to *V. faba* corresponded to a much higher plasma concentration of L-dopa.^33^ These scientific documents exhibit that the consumption of *V. faba* can lead to a considerable enhancement in plasma concentration of L-dopa along with improvement in motor proficiency in PD patients. The protective effects of *V. faba* on PD were summarized in Table 2.

**Table 2:** The protective effects of *V. faba* on PD

**Doses used**	**Model of study**	**Effects**	**Ref.**
250 g cooked	Human	Improvement of the clinical signs in PD patients and enhancement of plasma level of L-dopa	32
200 g + carbidopa		Increase in duration of motor response along with enhancement in plasma concentration of L-dopa	333

### Nigella sativa L.

**Figure 3:**
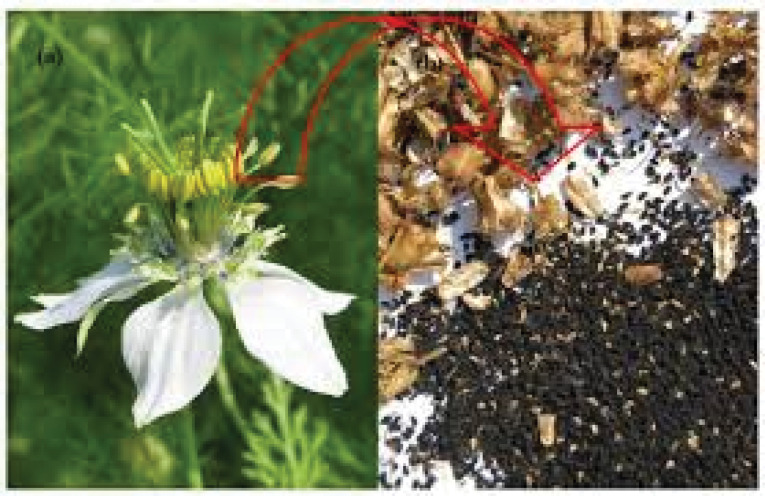
*Nigella sativa* L.

*Nigella sativa* is one of plant species of Ranunculaceae family which is flourished in the most part of world (Figure 3). The seeds ofthis medicinal plant are added as a food additive and spice to Persian foods including bread, pickle and salads.^34^ Ethanolic extracts of *N. sativa* (200 and 400 mg/kg) remarkably attenuated catalepsy in rats group extract compared to those of treated by chlorpromazine (CPZ) (3 mg/kg i.p.). *N. sativa* extracts also significantly decremented the amount of lipid peroxidation and level of nitrite and augmented glutathione vis-à-vis CPZ-treated group.^35^

The protective effects of* N.* sativa and its effective ingredient, TQ, on central nervous system (CNS) disorders such as neurotoxicity, epilepsy, PD and AD have been reviewed.^36,37^ *N. sativa* and TQ have been also documented to possess the anti-inflammatory, anti-oxidant, anti-cancer, anti-genotoxic and hepato-protective effects.^38-40^ It has been also reported that *N. sativa* oil can protect nervous system against Aβ-caused neurotoxicity through antioxidant effect in primary cerebellar neurons in rats^41^ as well as its effect on learning and memory.^42^ Oral administration of *N. sativa* hydroalcoholic seeds extract (100 and 200 mg/kg) has been reported to improve perphenazine-induced muscle rigidity score in mice, while in animals treated with 50 mg/kg of this plant extract had no any significant effect on this parameter compared to control group.^43^

Administration of *N. sativa* capsules (500 mg) twice daily for 9 weeks in 40 healthy volunteers increased attention, cognition and memory with respect to the placebo (500 mg) capsules-treated group.^44^ Similarly, in the other clinical study the effects of* N. sativa* capsules (500 mg) on healthy adolescent 14 - 17 years old (n=48) once daily for one month were evaluated. All healthy adolescent were managed for mood, cognition and anxiety with the relative tests in the start and the end of the study. *N. sativa* capsules (500 mg) decreased anxiety, stabilized mood and modulated cognition at the end of study.^45^ Therapeutic effect of TQ on behavioral, cellular changes and oxidative stress biomarkers in 6-OHDA-induced Parkinson’s rat model was assessed. Pretreatment with TQ (5 and 10 mg/kg) reduced the level of malondialdehyde (MDA) and prevented the loss of substantia nigra pars compact neurons.^46^ The neuro-protective effects of TQ against 1-methyl-4-phenylpyridinium (MPP^+^) and rotenone caused-toxicity in cell culture of dopaminergic cells of mouse mesencephalic were also investigated.Treatment withTQ (0.1 and 1 μM) saved about 25% of dopaminergic cultures (THir neurons) against MPP^+^-induced cell death. Furthermore, TQ (0.1, 1 and 10 μM) in a dose-dependent manner protected the THir neurons respectively 65%, 74% and 79% against rotenone-induced toxicity.^47^ The results of other study suggested that TQ had neuro-protective potential and could exert as a promising therapeutic agent to reduce the risk of developing of AD and other neurodegenerative disorders of the CNS such as PD.^48^

Carvacrol (CARis a monoterpenic phenol which is found in many aromatic plants such as *N. sativa*. Anti-inflammatory and antioxidant effects of carvacrol have been showed previously.^49,50^

In a study, CAR (40 mg/kg) induced a considerable neuro-protective effect against the unilateral 6-OHDA-caused Parkinson model in male mice. This protective effect was associated with down-regulation of caspase -3.^51^ Intrapretoneal adminstration of CAR (12.5 or 25 mg/kg) in a reserpine (RES)-triggered rat model of PD could prevent the increase in catalepsy behavior and number of vacuous chewing movements, but could not revert the decreased locomotor activity in open field test. Furthermore, CAR impeded the decrease in tyrosine hydroxylase (TH) immunostaining induced by RES in the substantia nigra pars compact and dorsal striatum.^52^

Pretreatment with CAR (10 mg/kg/d) also attenuated the neurotoxicity effect of 6-OHDA in hemi-Parkinson rat model. In this study, CAR significantly decreased the levels of MDA and nitrite content and enhanced catalase activity in midbrain. These results indicated that protective effect of CAR probably was mediated through ameliorating oxidative stress.^53^ Administration of CAR (25, 50 and 100 mg/kg, ip) for 6 weeks in 6-OHDA- lesioned rat model of PD ameliorated memory deficits. The results showed that CAR did not affect the rotation and hyperalgesia in lesioned rats. Based on results, CAR in mentioned doses also could not restore the decreased level of total thiol in the striatum of animals treated with 6-OHD.^54^ The protective effect of *N. sativa* and its components on PD were shown in Table 3.

**Table 3:** The protective effect of *N. sativa* and its components on neurotoxicity andParkinson's disease

**Plants /component**	**Dose**	**Model of study**	**Effects**	**Ref.**
*Nigella sativa*	200 and 400 mg/kg	Rat	Attenuation of catalepsy, reduction of the amount of lipid peroxidation and nitrite level, augmentation of glutathione content	35
	100 and 200 mg/kg	Mice	Improvement of perphenazine-induced muscle rigidity score	43
	500 mg/kg	Human	Enhancement of attention, memory and cognition	44
	500 mg/kg	Human	Decrease of anxiety, stabilization of mood and modulation of cognition	45
TQ	5 and 10 mg/kg	Rat	Reduction of MDA level and prevention of the loss of substantia nigra pars compact neurons	46
	0.1 and 1 μM	THir neurons	Protection of dopaminergic cultures against MPP^+^ and rotenone-induced cell death.	47
Carvacrol	40 mg/kg	mice	down-regulation of caspase -3	51
	12.5 or 25 mg/kg	Rat	Prevention of increase in catalepsy behavior and number of vacuous chewing movement and mitigation of HT in RES-triggered rat model of PD in the substantia nigra pars compact and dorsal striatum	52
	10 mg/kg/d	Rat	Attenuation of neurotoxicity, decrement of MDA and nitrit and enhancment of catalase activity in 6- OHDA-caused hemi-Parkinson rat model	53
	25, 50 and 100 mg/kg	Rat	Amelioration of memory deficits 6-OHDA- lesioned rat model of PD	54

### Crocus sativus L.

**Figure 4:**
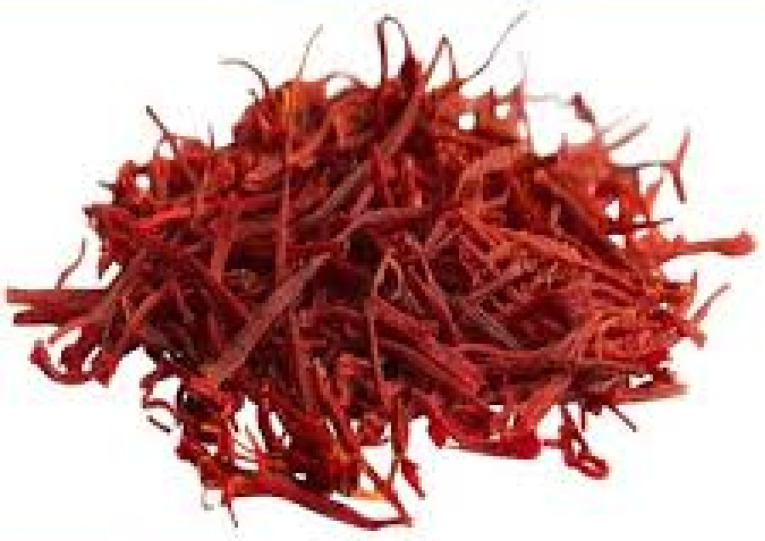
*Crocus sativus* L.

*Crocus sativus* or saffron from the Iridaceae family was cultivated in many countries including Iran, Turkey, Afghanistan and Spain (Figure 4).^55^ *C. sativus* and its constituents are used to treat cognitive disorders and some neural disorders. This herb is also used as smooth muscle relaxant agents in Iranian traditional medicine.^56-58^ Saffron and its components have been suggested to have useful effects in neurodegenerative disorders in animal studies.^59^ The protective effect of *C. sativus* on dopaminergic cells in the substantia nigra pars compact and retina in a mouse model of 1-methyl-4-phenyl-1,2,3,6-tetrahydropyridine (MPTP)-induced acute PD was studied. Administration of *C. sativus* (0.01% w/v) in drinking water saved dopaminergic cells of the substantia nigra pars compact and retina from MPTP-induced injury in mice.^59^

Formation and accumulation of toxic amyloid structures can result in induction of neurodegenerative disorders including PD and AD. Crocin and safranal, two main components of *C. sativus*, have been reported to inhibit fibrillation of apo-α-lactalbumin causing neuronal damage under amyloidogenic conditions.^60^ Treatment with C. *sativus* (50 mg/kg) has been also shown to prevent the development of PD in lead (Pb)-induced damage in nervous system. This effect was associated with the increase of TH level in various brain areas including substantia nigra compacta, locus coeruleus, dorsal striatum and medial forebrain bundle of mice.^61^

The oral administration of crocin (30 mg/kg) on MPTP-stimulated Parkinson model in male BALB/c mice after 15 days treatment resulted in the increment of staying time on the wire in the hanging test. Treatment with crocin also improved MPTP-induced cell death in the substantia nigra compacta of mice.^62^ Intraperitoneal administration of crocin (30 and 60 mg/kg/d) could also diminish the thiobarbituric acid reactive substance and nitrite levels in the hippocampus after 6 weeks in 6-OHDA model of PD in rats.^63^ Intraperitoneal injection of *C. sativus*-extracted crocetin (25, 50 and 75 µg/kg) has been also indicate to have neuro-protective effects in Parkinsonism rat model caused by 6-OHDA. In this study, the enhanced level of antioxidant indicators and dopamine content was reported.^64^ Crocetin also has been shown to rescue substantia nigra compacta against thiobarbituric acid when it was intraperitoneally infused.^65^ The Results of research studies indicated that crocin derived from *C. sativus* (10 µM) protects the PC12 cells against MPP^+^–caused injury and can improve cytotoxicity related to endoplasmic reticulum.^66^ The protective effects of *C. sativus* and crocin on neurotoxicity and PD has been condensed in Table 4.

**Table 4:** The protective effects of *C. sativus* and crocin on neurotoxicity and Parkinson's disease

**Plants /Component**	**Doses**	**Model of study**	**Effects**	**Ref.**
*C. sativus*	0.01% w/v	Mice	Protection of dopaminergic cells in the substantia nigra pars compact and retina against MPTP-induced injury	59
	50 mg/kg	Mice	Prevention of the development of PD in Lead Pb -induced damage in nervous system, increase of TH level in various brain areas including substantia nigra compacta, locus coeruleus, dorsal striatum and medial forebrain bundle	61
Crocin	30 mg/kg	Mice	Increment of staying time on the wire in the hanging test and prevention of cell death in the substantia nigra compacta in MPTP-stimulated Parkinson model	62
	30 and 60 mg/kg/d	Rat	Decrease of thiobarbituric acid reactive substance and nitrite level in the hippocampus after 6 weeks in 6-OHDA model of PD	63
	25, 50 and 75µg/kg	Rat	Enhanced level of antioxidant indicators and dopamine content in Parkinsonism model caused by 6-OHDA	64

### Curcuma longa

**Figure 5:**
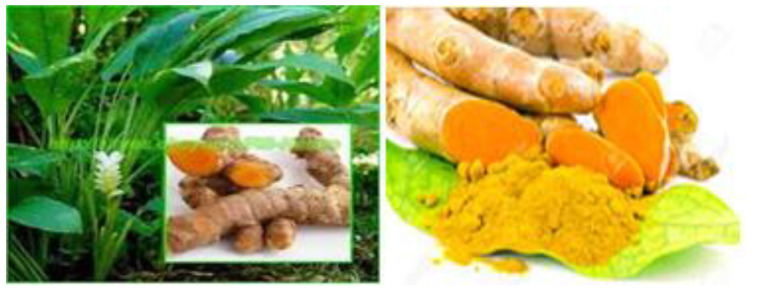
Curcuma longa.

*Curcuma longa*, turmeric, is widely grown and cultivated as spice in the south-east Asian countries (Figure 5).^67^ This medicinal plant possesses natural polyphenol and non-flavonoid modulating oxidative damage of nervous system^68^ and other body organs^69,70^ *C. longa* has been also realized to have several pharmacological effects including anti-inflammatory and anti-cancer.^71^ In a study, the aqueousextractof *C. longa* (560 mg/kg) significantly could inhibit the activity of dopamine metabolizing enzyme, monoamine oxidase A (MAO_A_), in the brain of mice.^72^ The *C. longa* extract (0.001, 0.01, 0.05, 0.1, 0.2 and 0.4 mg/mL) also ameliorated salsolinol-induced toxicity in human neuroblastoma cells (SH-SY5Y cells), reduced mitochondria-derived ROS and down-regulated caspase 3 activity.^73^ It has been reported that water soluble extract of curcumin (50-200 mg/kg p.o.) increased serotonin and dopamine level in the brain tissues and dose 50 mg/kg of it enhanced the antidepressant-like effect of classical antidepressants drugs in mice.^74^ Curcumin (5, 10 and 20 mg/kg) increased the content of monoaminergic neurotransmitters including norepinephrine and dopamine in hippocampal tissue. Furthermore, curcumin obviously up-regulated the expression of derived neurotrophic factor (BDNF), TrkB, and phosphatidylinositide 3-kinases (PI3K) in hippocampal tissue.^75,76^ Administration of curcumin at doses 50, 100 and 200 mg/kg improved cognitive deficits and mitochondrial dysfunction in mice.^77^ Intraperitoneal injection of curcumin (50 and 100 mg/kg,) improved neurological deficits and increased the number of NeuN-labeled neurons in the ischemia reperfusion in rats.^78,79^ Immunohistochemistry results showed that curcumin (0.1, 1 and 10 µM) inhibited p-IRE1α, p-PERK and NLRP3 expression in hippocampus CA1 region of rtas.^80^ Li et al also suggested that curcumin exerts protective effects on rats brain against cerebral ischemia- reperfusion injury through increasing neuron survival, reducing inflammatory cytokine production and activating JAK2/STAT3 signaling pathway.^81^ It has been documented that curcumin (5 and 10 μM) restored malic impact of OxyHb on livability of primary cortical cells and decreased their apoptosis.^82^ Wang et al reported that curcumin (10 μm) significantly inhibited 6-OHDA-induced NFκB transcription in the MES23.5 cells and inhibited ROS intracellular accumulation.^83^ Curcumin has been also reported to improve nitric oxide (NO)-mediated degeneration in PC12 cells.^84^ Curcumin (500nM) also could inhibit the MAO_B_ activity with both the competitive and noncompetitive inhibition. This effect of curcumin was comparable with the effect of selegiline as a MAO_B_ inhibitor.^85^ According to these results, curcumin can be considered as a possible cause for inhibiting MAO_B_ and be used in the treatment of PD and other neurological disorders. Oral administration of curcumin (100 mg/kg) ameliorated muscular strength in rotenone-induced motor deficits in rats. Treatment with curcumin also drastically increased the falling time of rats from inverted screen compared to treated group with rotenone. Curcumin also significantly improved stride length of forelimb, hind limb; hind base and paw overlapping in rats. Curcumin pretreatment significantly attenuated the decreasing effect of rotenone on function of dopaminergic system in striatum via increasing the level of dopamine and dihydroxyphenylacetic acid. In addition, the glutathione levels of treated rats with curcumin significantly increased with respect to those of rotenone group.^86^ The protective effects of C. longa and curcumin on PD were compacted in Table 5.

**Table 5:** The protective effects of *C. longa* and curcumin on neurotoxicity and PD

**Plants /Component**	**Doses**	**Model of study**	**Effects**	**Ref.**
*C. longa*	560 mg/kg	Mice	Inhibition of the activity of dopamine metabolizing enzyme, MAO_A_ in the brain	72
	0.001-0.4 mg/ml	SH-SY5Y cells	Amelioration of salsolinol-induced toxicity, reduction of mitochondria-derived ROS and down-regulation of caspase 3 activity	73
Curcumin	50-200 mg/kg	Mice	Increase of serotonin and dopamine levels in the brain tissues and enhancement of the antidepressant-like effect of classical antidepressants drugs	74
	5, 10 and 20 mg/kg	Rat	Increase of monoaminergic neurotransmitters content and up-regulation of derived neurotrophic factor BDNF , TrkB, and phosphatidylinositide 3-kinases PI3K expression in hippocampal tissue	75,76
	50, 100, 200 mg/kg	Mice	Improvement of cognitive deficits and mitochondrial dysfunction	77
	50 and 100 mg/kg	Rat	Improvement of neurological deficits and increase the number of NeuN-labeled neurons in the ischemia reperfusion	78.^79^
	0.1, 1 and 10 µM	Rat	Inhibition of p-IRE1α, p-PERK and NLRP3 expression in hippocampus CA1 region	80
	5 and 10 μM	Cortical neurons	Improvement of cell viability and decreased neuronal apoptosis	82
	10 μm	MES23.5 cells	inhibition of 6-OHDA-induced NFκB transcription and ROS intracellular accumulation	83
	500nM	Rat	Inhibition of the MAO_B_ activity with both the competitive and noncompetitive	85
	100 mg/kg	Rat	amelioration of muscular strength, increase of falling time, improvement of stride length of forelimb, hind limb; hind base and paw overlapping in rotenone-induced motor deficits. Attenuation of the decreasing effect of rotenone on *glutathione* ***level*** and function of dopaminergic system in striatum via increasing the level of dopamine and dihydroxyphenylacetic acid.	86

## Conclusion

Our review narrates an overview of therapeutic properties of medicinal plants and their ingredients on PD. The experimental and clinical data emphasize that neuro-protective effect of medicinal plants including *M. pruriens, V. faba, N. sativa* and * C. sativus* mainly are mediated via reduction of oxidative stress and neuro-inflammation resulting in the induction of PD. In addition, a part of anti-Parkinson properties of these plants can be attributed to the inhibition of MAOs and to modulate the content of neurotransmitters such as dopamine, norepinephrine and serotonin in the substantia nigra.

## Ethical Issues

Not applicable.

## Conflict of Interest

The authors declare that there is no conflict of interest regarding the publication of this paper.
